# Assessing the temporal stability of surface functional groups introduced by plasma treatments on the outer shells of carbon nanotubes

**DOI:** 10.1038/srep31565

**Published:** 2016-08-10

**Authors:** Andrea Merenda, Elise des Ligneris, Kallista Sears, Thomas Chaffraix, Kevin Magniez, David Cornu, Jürg A. Schütz, Ludovic F. Dumée

**Affiliations:** 1 Deakin University, Geelong, Institute for Frontier Materials, 3216 VIC, Australia; 2 CSIRO Manufacturing, Clayton - 3149 VIC, Australia; 3 Institut Européen des Membranes, IEM, UMR-5635, Université de Montpellier, ENSCM, CNRS, Place Eugène Bataillon, 34095 Montpellier cedex 5, France; 4 CSIRO Manufacturing, Waurn Ponds - 3216 VIC, Australia

## Abstract

Plasma treatments are emerging as superior efficiency treatment for high surface to volume ratio materials to tune functional group densities and alter crystallinity due to their ability to interact with matter at the nanoscale. The purpose of this study is to assess for the first time the long term stability of surface functional groups introduced across the surface of carbon nanotube materials for a series of oxidative, reductive and neutral plasma treatment conditions. Both plasma duration dose matrix based exposures and time decay experiments, whereby the surface energy of the materials was evaluated periodically over a one-month period, were carried out. Although only few morphological changes across the graphitic planes of the carbon nanotubes were found under the uniform plasma treatment conditions, the time dependence of pertinent work functions, supported by Raman analysis, suggested that the density of polar groups decreased non-linearly over time prior to reaching saturation from 7 days post treatment. This work provides critical considerations on the understanding of the stability of functional groups introduced across high specific surface area nano-materials used for the design of nano-composites, adsorptive or separation systems, or sensing materials and where interfacial interactions are key to the final materials performance.

The nature of the temporal stability of functional groups introduced across the surface of nano-materials is critical to the development of advanced materials for applications where surface-specific interactions or reactions determine performance. The density, distribution and type of functional groups introduced on the surface of nano-particles will provide synergistic effects to control interfaces across nano-composites, but also interactions with probe molecules during sensing or catalysis reactions, and affect separation performance if used as adsorbent or membrane materials.

Specifically, the surface functionalization of sp^2^ hybridized materials, such as graphene sheets or carbon nanotubes (CNTs), represents a challenge due to the inherent crystallinity and chemical stability of these unsaturated materials^1^,^2^. Approaches to finely tune the surface coverage and position of functional groups across the surface of graphitic planes have been developed to modify the physico-chemical properties of these materials at the nanoscale. The aim of such treatments is to facilitate specific interactions that prevent agglomeration during processing and incorporate CNTs uniformly into mixed matrix composite materials^3^,^4^,^5^. Other important aspects are concerned with increasing structural interactions between graphitic materials^6^,^7^, or enhancing surface-bound properties of materials in relation to plasmons or general adsorption^7^,^8^. The natural hydrophobicity of native graphite and graphene is due to the intrinsically non-polar structure consisting of aromatic ‘honeycomb’ sheets, which consequently show little affinity to highly polar molecules such as water^9^,^10^. Thus the purpose of the graphene-based materials chemistry in this regard is to enhance the scope and range of applications by either improving the chemical interaction potential or establishing covalent bridges to the surrounding matrix^11^,^12^,^13^,^14^.

Pathways to chemically modify graphene based materials are aiming to either open aromatic rings of sp^2^ hybridized structure or react with chemical groups attached to defect sites on graphitic planes. Types of treatment include chemical reactions in solution as well as exposure to sources of radiation (such as x-ray, gamma ray or ultra violet), reactive gases like ozone and radicals from plasmas^15^,^16^,^17^. Solution reactions are by far the most wide-spread of all surface-bound functionalization routes and are typically governed by fast kinetics of reaction. However, practical issues related to constraints in storing CNT suspensions due to particle agglomeration, the colloidal stability as a function of the pH or the functionalization of CNT are limiting potential applications to a relatively narrow range of surface modifications^10^,^18^. Moreover, due to the highly reactive nature of conditions required to apply these treatments, e.g. through the use of chemicals such as H_2_SO_4_ or HNO_3_, significant damage is often introduced to the sidewalls and tips of CNTs treated in this way^16^,^19^,^20^. In fact, attaching covalently-bound functional groups to CNT or graphene structures will always lead to local defect formation and re-arrangement of the graphitic lattice prior to orbital de-hybridization when planar sp^2^ bonds are transformed to sp^3^ bonds. The grafting of complex macro-molecules via solution modification typically requires a series of reactions, involving multiple cleaning and purification procedures, to process graphene materials, leading to low reaction yields^21^,^22^. On the other hand the high penetration power of radiation-based reactions, such as x-ray or gamma ray irradiation, allows treatments to be performed in the gas phase that are not limited to the material surface. Nonetheless they were shown to homogeneously functionalize many layers of graphene tens of microns thick^23^,^24^,^25^. Radiation is extremely efficient at altering the crystalline structure of graphitic planes and interact with defects within the structure to promote simple forms of functionalization, such as grafting of carboxylic, amine or hydroxyl groups. However, the capacity of this technique to graft more complex functional groups is limited to reagents which can be brought into physical contact with the target sites^23^,^26^,^27^. The same applies to UV, ozone and plasma, however these treatments provide extensions to the range of surface modifications available for graphene materials^7^,^28^,^29^. Reactive gas plasma treatments and polymerization by plasma enhanced chemical vapour deposition (PECVD) offer the possibility to chemically and morphologically tune material surfaces by introducing new functionalities through a process that is friendly to the environment, quick and highly efficient.

Recent work on plasma treatments have shown that complex series of functionalization, grafting or coating steps can be applied to enhance surface properties of meta-materials in terms of electrical conductivity, surface abrasion resistance and surface energy^30^. Another fundamental challenge is related to controlling plasma conditions in order to vary the impact the process has on the crystallinity of the material and the level of damage it inflicts on the structure, possibly altering texture and molecular order at the nano or meso scale^31^. It was previously shown that the number of defects generated across highly crystalline materials was primarily dependent on plasma excitation power, the partial pressure of the feed gas and treatment time due to the effect these parameters have on plasma density, electromagnetic radiation and flux of ionized matter^32^,^33^,^34^. These findings imply that plasma parameters need to be finely tuned to obtain the most suitable result for a certain target application. The reactivity of surfaces after plasma treatment is still being debated, since the stability of functional groups introduced across CNTs upon plasma treatment has not been discussed to date and it is essential to evaluate the long-term efficiency of this fabrication route towards a possible scale-up. The levels of hybridization of graphitic plans, and the reactivity of vacancies generated upon activation may lead to a number of functionalization pathways depending on the type of species present within the plasma glow. Stochastic generation of radicals within the plasma would lead to the formation of both sp3 bonds which may rehybridize into sp^2^ states. It has been shown that even an inert feed gas, such as Argon, has the capacity to produce oxygenated functionalities when reactive chemical surface groups generated by the plasma treatment are brought in contact with oxygen from the air^35^. Reported applications for plasma treatments applied to graphene based materials include tuning interactions of treated material with the environment, which favor the attachment of certain molecular groups over others, as well as the etching and cleaning of surfaces^36^,^37^,^38^. Several studies have been carried out to demonstrate how molecular reactants such as nitrogen, ammonia or oxygenated gases and vapours can be used for plasma treatment^39^,^40^,^41^. The interaction of the free radicals generated by the plasma with the surface of graphene materials has, however, not been studied to date. The assessment of the long-term stability of surface groups generated by the plasma is critical to its role for manufacturing in general, where graphene sheets or carbon nanotubes are modified for the purpose of incorporating them into complex hierarchical materials^42^,^43^.

The aim of this work is to evaluate the lasting impact of plasma treatments on carbon nanotube bucky-papers (BPs). This study was performed in order to gauge the reactivity of the CNTs and hence that of other graphene based materials, to different plasma environments (oxidative, reductive and neutral) and to quantify the density and type of functional groups that are created by the treatment. It also seeks to quantify the damage caused by the plasma treatment to the crystalline structure of the CNTs as a function of specific conditions. X-ray photoemission spectroscopy (XPS), Raman spectroscopy and Photoelectron Spectroscopy in Air (PESA) measurements were performed on the treated samples to monitor the degree and type of functionalisation, structural damage to the CNTs, and the surface work function, respectively. The long term stability of functional groups introduced onto the CNTs was investigated by characterizing the samples at set periods up to 1 month post treatment. The different plasma treatments were compared, with results illustrating the potential of pertinent plasma treatment techniques for mass production of graphene based materials exhibiting versatility and characterized by excellent chemical stability.

## Results and Discussions

**Figure 1:**
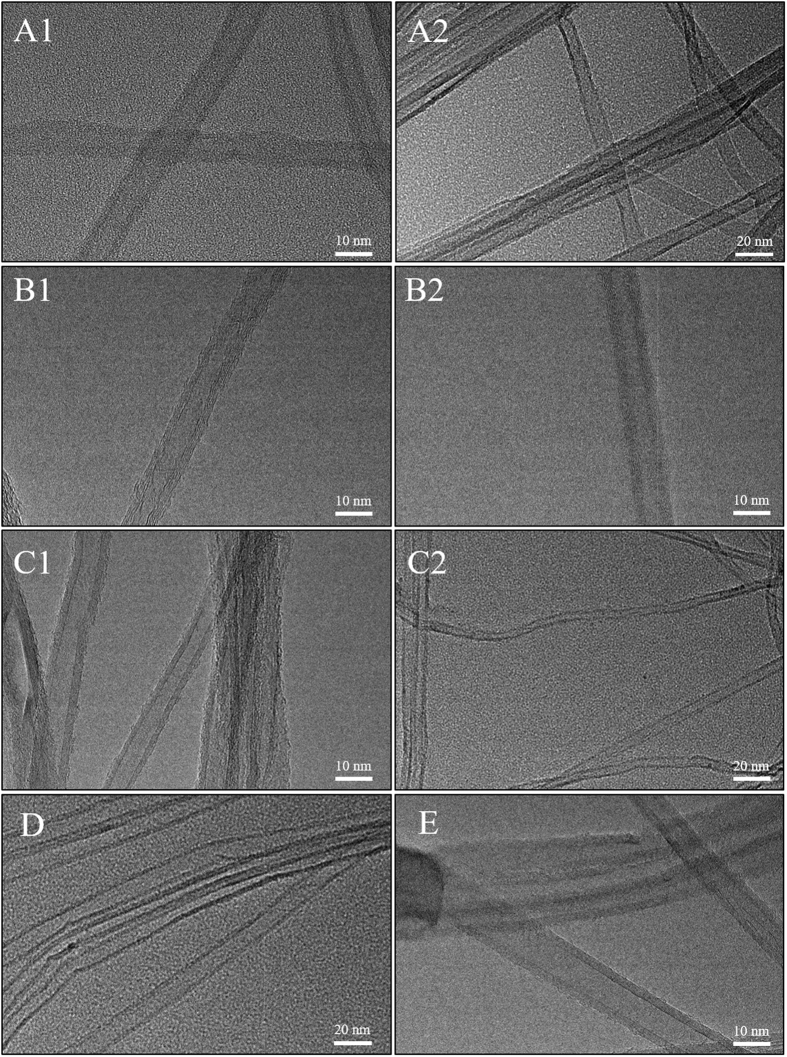
Transmission Electron Micrographs (TEMs) of CNTs under different conditions: reference (**A1,A2**), plasma treated with O_2_/Ar (**B1,B2**) for 1 min and 30 min respectively, plasma treated with H_2_/Ar (**C1,C2**) for 1 min and 30 min respectively, CO_2_ 5 min (**D**) and Ar 30 min (**E**).

**Figure 2:**
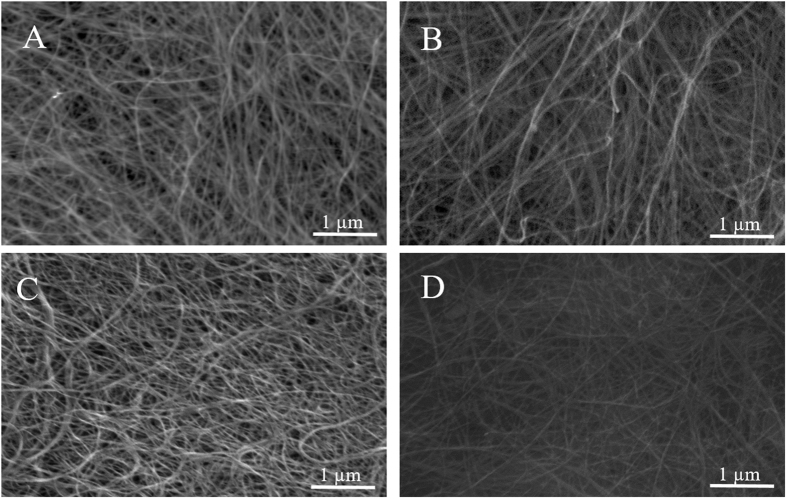
Scanning Electron Micrographs (SEMs) of plasma treated BPs for 30 min with O_2_/Ar (**A**), H_2_/Ar (**B**), CO_2_ (**C**) and Ar (**D**) respectively.

The impact of the plasma treatments on the morphology of the CNTs was investigated by evaluating the integrity of the crystalline graphitic walls in terms of the presence of damage. The structure of the untreated CNTs were compared to that of the plasma modified samples across the TEMs in Fig. 1 and Figure S1. Although the outer wall profiles seemed to be affected by the treatment, showing slight breakages all along the tubes, no severe damage could be seen up to 30 min of plasma treatment, regardless of the nature of the gas feed. Consequently, the plasma conditions could be considered gentle enough to preserve the physical structure of the samples even at longer treatments while the nature of the gas as reducing, oxidizing or inert, cannot be directly related to these effects since the micrographs do not show any significant difference across the series. The TEM analysis was confirmed by a long range SEM analysis as shown in Fig. 2. The surface modifications did not generate major physical damages on the pristine substrate presented in Figure S2, suggesting that the material crystallinity was largely unaffected by the plasma treatments. Furthermore, no evidence of a possible correlation between the feed gas and the final physical structure can be inferred since apparently there is no difference across the series of different gas. Interestingly the exposure duration does not seem to have an impact in terms of damage as no remarkable differences can be assumed comparing shorter treatments, presented in Figure S3, with the longest.

**Figure 3:**
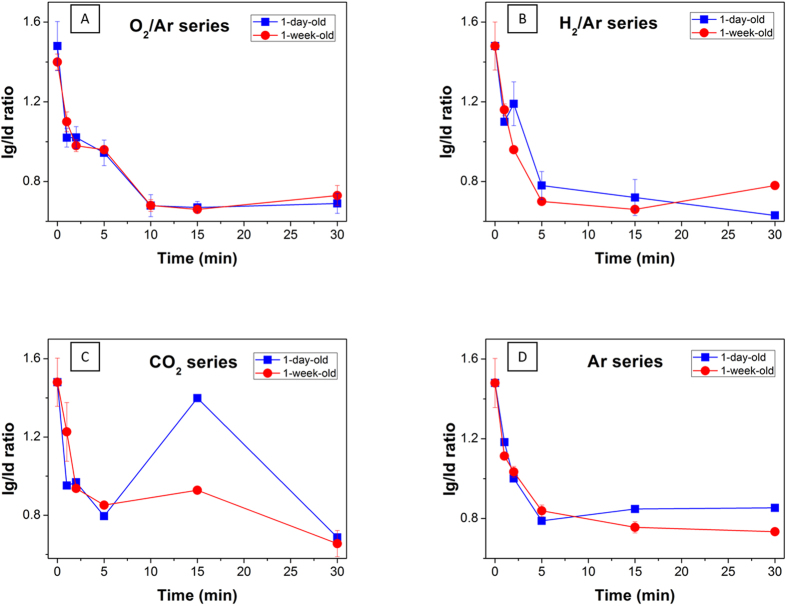
Raman Ig/Id ratio of the O_2_/Ar series (**A**), (**B**) H_2_/Ar, (**C**) CO_2_ and (**D**) Ar.

As shown in Fig. 3, the level of disorder associated with the structure and the introduction of defects was evaluated by comparing Raman spectra across various series of different plasma durations as well as different gases. The nature of the disorder which may have been introduced by the plasma treatments and the quantification of the amount of damage, were related to the main plasma parameters including plasma gas and duration. Raman scattering on functionalized CNTs are therefore performed to evaluate the intensity of the G- and D-bands of the Raman spectra to quantify the amount of defects generated in the lattice. A quantitative analysis on the defects occurring across graphitic plans may be assessed typically by analyzing the I_g_/I_d_ ratio corresponding to the G and D bands at 1583 cm^−1^ and 1352 cm^−1^ respectively^44^. The G-band represents the tangential stretching vibration of the in-plane C-C bonds in the graphitic structure while the D-band is a double-resonance Raman mode generated by the inelastic scattering of a phonon and elastic scattering induced by a defect^45^. While the G-band is taken into account to discuss the graphitic lattice of the CNTs, the D-band is attributed to the presence of disorder in graphitic materials. All the spectra were normalized on the G’ band occurring at 2705 cm^−1^, the variation of the G and D bands over time duration for O_2_/Ar series is reported in Figure S4 as an indicative example. In addition to providing evidence of the introduction of functional groups, the I_g_/I_d_ ratio may be used to quantify the amount of amorphous sp^3^ carbons generated from damage on the walls of the CNTs^46^ although the technique itself cannot provide a definitive qualitative measure for functional groups that are present. The investigation was conducted via a series of plasma treatment durations, while the samples were analysed within 6 h of treatment to minimize effects from oxidation in air. For these series, a significant drop of the I_g_/I_d_ ratio between the pristine BP and the 1 min treated sample is reported for each feed gas. The ratio decreases by approximately 31%, from 1.48 to 1.02 for the O_2_/Ar series and by 36% drop at 0.92 for the CO_2_ series. The weakest plasma impact was obtained initially for Ar, where I_g_/I_d_ is dropping to 1.18 under comparable conditions. Interestingly, the ratio is plateauing for longer treatment durations in the vicinity of 0.75 for any of the different reactant gases. The pronounced initial change of the I_g_/I_d_ ratio can be interpreted as that the plasma was contributing significantly to the introduction of defects and possibly new functional groups within the first few minutes of treatment. This can be further confirmed by the analysis of the Raman spectra of each feed gas, reported in Figure S, where a shoulder at 1620 cm^−1^ defined as D’ band arises at the 5 min mark across the series^45^,^47^ along with the introduction of new functional groups. This result is in good accordance with previous studies^48^,^49^ and can be used to confirm the increased disorder due to functionalization.

The surface lattice eventually reaches a saturation level whilst a longer exposure to plasma produces no dramatic introduction of new functional groups. Interestingly, the H_2_/Ar and the pure Ar series, corresponding to neutral and reductive gases lead to I_g_/I_d_ ratios of 1.1 and 1.18 respectively after 1 min of exposure, suggesting that the nature of the gases involved is critical to control the level of damage introduced on the lattice for short treatment durations. This is further confirmed by the fact that even though all the series seem to reach a saturation level, the plateau is still at different I_g_/I_d_ ratios spanning from 0.77 for the Ar series to 0.63 for the H_2_/Ar. Consequently, it may be inferred that the effect of exposure duration on surface damage is relevant only for shorter treatments and becomes insignificant when saturation is reached. In that scenario it can be assumed that the nature of the reactant gas is what determines the nature of the physical damage.

During the plasma functionalization, different molecules may be formed following the proposed mechanisms described in former studies^35^,^50^,^51^, related to the presence of ions and radicals generated from the feed gas within the plasma glow. In the case of an inert gas, such as Ar, the activated species may interact as metastable positive ions along with the generated electrons with the surface scavenging reactive groups and creating more activated sites^52^. These sites, consisting initially of radicals generated on the surface, can therefore react with the active molecules present in the complex plasma atmosphere depending on the reactant gas used, leading to the formation of covalent bonds and resulting eventually in the grafting of chemical groups^51^. As for the oxygen plasma, the attack of the oxygen radicals for the gas considered, would likely lead to the formation of activated sites and then of C-O bonds on the surface of the BPs. Hydroxyl bonds are formed on stabilization by hydrogen atom transfer from the neighbors or on exposure to atmosphere after plasma treatment via interactions with humidity in the air and subsequent stabilization by hydrogen atom transfer from moisture, while carbonyl bonds are the consequence of an intramolecular reorganization of C-C bonds. Conversely the carboxylic groups are formed from active sites near previously generated carbonyl bonds after stabilization due to a proton transfer^51^.

**Figure 4:**
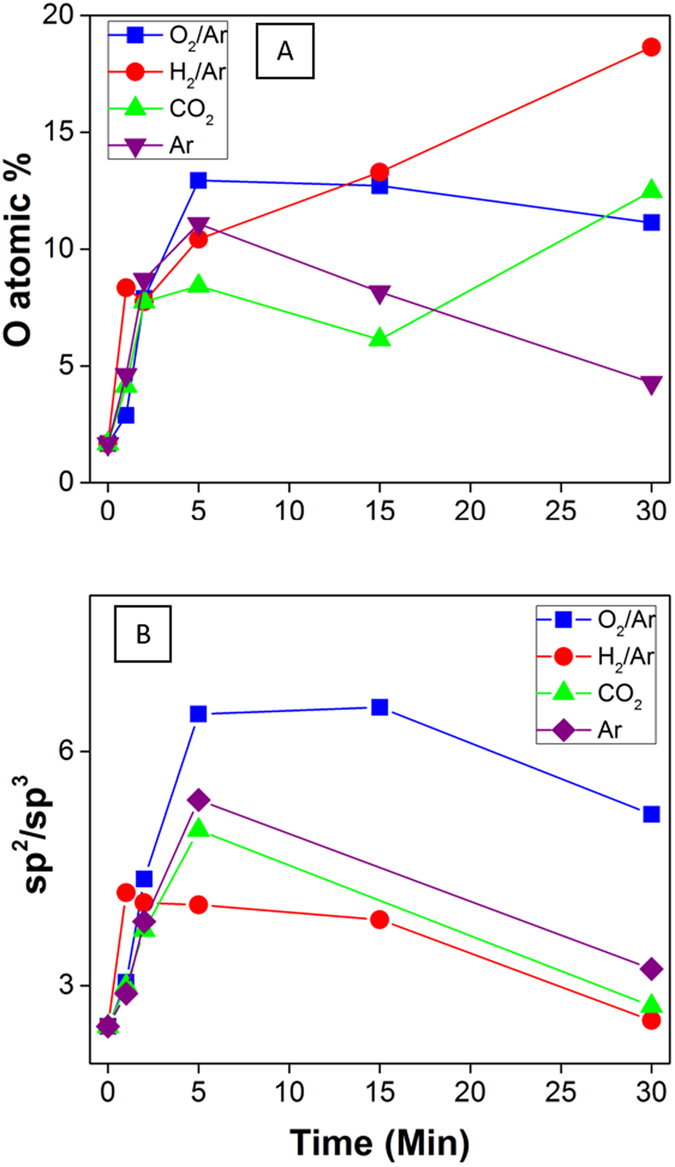
(**A**) Oxygen atomic concentration, (**B**) sp^2^/sp^3^ carbon ratio evaluated by X-ray photoemission spectroscopy (XPS) across the different series: O_2_/Ar (blue), H_2_/Ar (red), CO_2_ (green), Ar (purple) at different plasma treatment duration.

The nature of such changes in surface chemistry after plasma treatment is supported by results from XPS analysis. Figure 4A shows the evaluation of the oxygen (O) atomic concentration across the series calculated from the XPS survey showed in Figure S5 as well as the variation of sp^2^ and sp^3^ carbon which is relevant to assess the loss in graphitic structure. Interestingly, the main changes in O at% occurred within 5 min of plasma treatment, raising from 1.7 at% of the reference to 11.1 at% and 12.9 at% for the Ar and O_2_/Ar series respectively, representing the lowest and highest shifts. A similar oxygen content increase was observed from low-temperature thermal annealing under vacuum when the annealing time exceeded 2 h^53^. An increase in chemical activity was suggested as a possible cause for this sudden rise in the atomic concentration of oxygen, which occurred most likely when the sample was returned from vacuum to atmosphere. Whilst for the first 5 min the trend for each feed gas is the same with the observed remarkable increase in oxygen content, this increase was found to stop for longer treatments with the exception of the H_2_/Ar series for which the increase continued at a slower rate. It is assumed that the deviating trend for H_2_/Ar may be caused by further reaction pathways occurring between the activated CNT surfaces and oxygen in the air outside the plasma chamber and prior to storage in a dry environment. As observed for Ar-ion bombardment inside an XPS [54] or an ion gun^53^, the exposure to Ar plasma was reducing the atomic oxygen content for longer treatment times. Nonetheless, the analysis of the sp^2^ carbon suggests that the O_2_/Ar series is clearly the least costly in terms of lattice structural damage, showing a low 3% loss when compared to Ar, CO_2_ and H_2_/Ar which produced losses of 17%, 15% and 22%, respectively. These important results, considered in conjunction with those from the Raman analysis where a significant drop in the I_g_/I_d_ ratio was observed for all reactant gases in the first 5 minutes of treatment, suggest that for the O_2_/Ar series this drop is due to an actual introduction of functional groups containing oxygen, consuming amorphous sp^3^ carbon in the structure, rather than affecting the graphene sheets. This interpretation would be consistent with a stark drop in sp^3^ C for all reactant gases, while the sp^2^ C fraction for O_2_/Ar plasma remained essentially constant. Conversely, the H_2_/Ar plasma is reported to lead to the highest decrease in sp^2^ bonds with increasing treatment time while the sp^3^ structure is less affected when compared to the O_2_/Ar series, as shown in S6a,b. This result is consistent with the studies carried out on diamond-like carbon (DLC) where the H_2_ plasma exposure of thin films has been shown to etch away unsaturated bonds while increasing the sp^3^ content^54^.

The sp^2^/sp^3^ ratio was calculated, from the XPS data, for the series of samples. The sp^2^/sp^3^ ratio corresponds to ratio of the C=C to C-C carbon atoms. The graphitic walls of the pristine CNTs are solely composed of C=C bonds. However defects, either in the form of adduct groups, generated from a single C-C bond dissociation and corresponding to sp^3^ hybridized carbon atoms, or from multiple C-C bonds dissociation leading to functional groups generation. Stripping of carbon atoms from the main lattice may also generate either adduct defects or functionalization with oxygen rich groups, such as epoxy, carboxylate or hydroxyl^51^.

**Figure 5:**
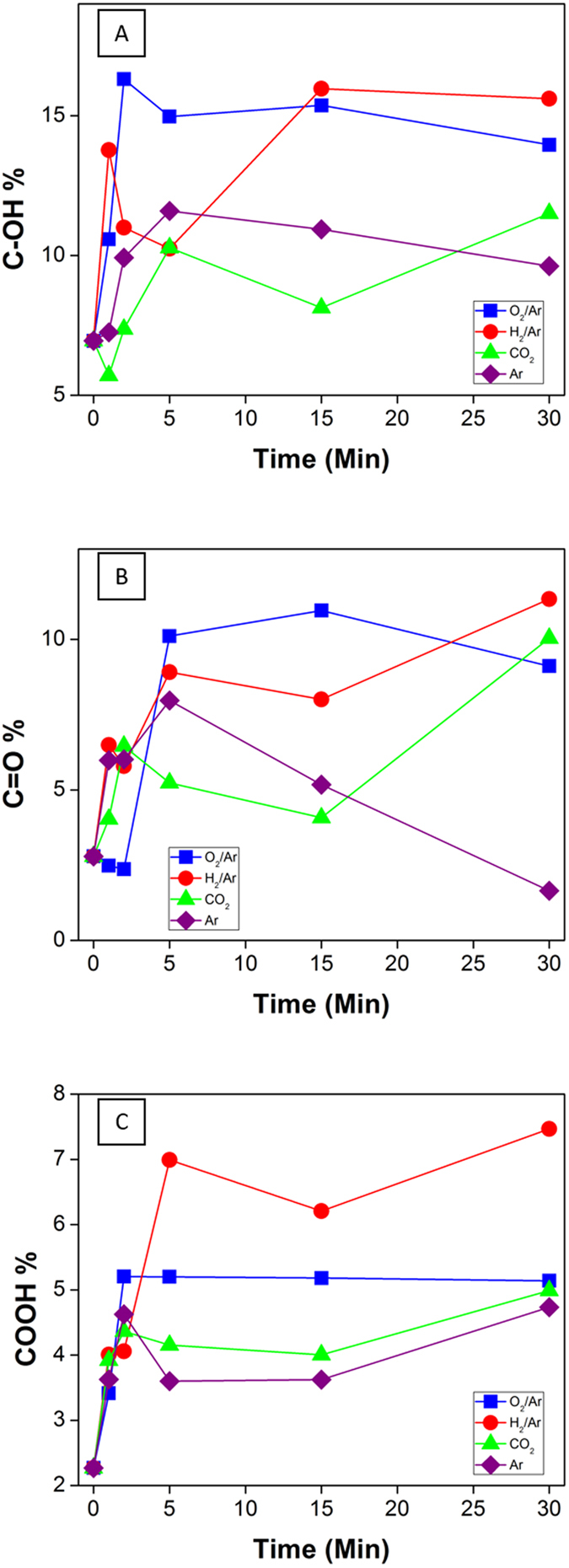
(**A**) relative content on the C1s spectra of C-OH (**A**), C=O (**B**), COOH (**C**) bonds evaluated by X-ray photoemission spectroscopy (XPS) across the different series: O_2_/Ar (blue), H_2_/Ar (red), CO_2_ (green), Ar (purple) at different plasma treatment time.

As seen in Fig. 4B, the sp^2^/sp^3^ ratio initially increased for short plasma durations, up to 5 min, prior to strongly decreasing for all plasma gases with longer plasma durations. The initial increase is attributed to the activation of the surface leading to a partial but rapid removal of sp^3^ hybridized atoms, known to be more reactive than sp^2^. Past 5 min, it is likely that excess energy input led to the breakage of bonds bridging sp^2^ carbons, which led to the sharp degree of functionalization as seen in Figs 4A and 5A–C. The oxygen content was in fact extremely well inversely correlated with the sp^2^/sp^3^ ratio and the amount of carboxylate and hydroxyl groups calculated from the XPS carbon peaks deconvolution. This result is in excellent agreement with previous calculations of bond energy dissociations and for CNT graphitic plans^55^. It therefore can be assumed that the rise in sp^3^ carbon shown for long treatment duration is due to the conversion of the sp^2^ structure with the formation of C-H functionalities, occurring only after a long exposure given the low concentration of hydrogen, 2%, as reported. The effective change is best gauged by comparing the H_2_/Ar result to pure Ar. In contrast, the absence of a reductive agent when O_2_/Ar is used leads to a more extensive reaction of amorphous carbon, consistent with the greater reactivity previously attributed to such sites in the literature. This assumption is confirmed in Figure S6a,b showing a significant drop in sp^3^ hybridization from 23.8 at% to 9.1 at% within the first 5 min for the O_2_/Ar series^34^.

Furthermore, an in-depth analysis was carried out on the XPS results to determine the type and extent of specific functional groups generated by the plasma treatment. The XPS data was deconvoluted into 5 Gaussian peaks centred at 284.5, 285.2, 286.2, 287.7, 288.9 and 291.2 eV which are assigned to sp^2^ carbon, sp^3^ carbon, C-OH, C=O, COOH, and π- π* respectively. The results of this analysis are given in Fig. 5 (π- π* transition in Figure S6c) and in Figures S7–S10, presenting the high resolution spectra of C1s for each series, and broadly confirm the chemical pathway previously discussed. To establish a correlation between the type of carbon-hybridization and surface functional groups on CNT it is necessary to take into account that hydroxyl groups are associated most likely with the sp^2^ hybridization of carbon and a lattice defect or an edge, while carbonyl and carboxylate groups are associated with sp^3^ hybridization. The XPS graphs reveal that the hydroxyl groups represent the largest fraction of oxygen rich groups with the O_2_/Ar series emerging as the most efficient reactant gas that produces this functionality with an absolute maximum at 16.3%. The generation of hydroxyl groups is furthermore consistent with a minimal change in sp^2^ hybridization observed for this reactant gas. Interestingly, as already reported for the Raman scattering and the atomic elemental content, all the series saturate at longer treatment durations with the most significant changes occurring within the first 5 minutes of exposure. Oxygen surface saturation is therefore taking place across the surface when the sample is exposed to air after treatment, and minor changes between series are probably due to a re-arrangement of the graphitic walls from and with the significant amount of remaining activated sites. Carboxylic groups were found more predominantly with the H_2_/Ar treatments plateauing at 6.9% while the O_2_/Ar treatment clearly shows a surface saturation, plateauing at 5.2% after a short exposure of 2 min to the plasma environment. Interestingly, the H_2_/Ar gas also shows the most significant drop in sp^2^ carbon, down to an absolute minimum of 45.8% which can be correlated either to reduction reactions between the hydrogen radicals^56^ or to the breakage of C=C bonds by the H^+^ ions^54^,^57^ or H radicals which, conversely, stabilizes the sp^3^ C-C content. These results match previous studies where H_2_ plasma treatments were found to drastically reduce the electrical conductance upon treatment, with the loss of C=C bonds^56^,^58^. The decrease in π- π* transition content, shown in Figure S6c, which exhibits an sp^2^ satellite peak^59^, supports the analysis in terms of loss in sp^2^ hybridization particularly relevant for the H_2_/Ar series, as discussed. The lowest rates of reactions were obtained for the Ar gas with only a 14.3% final oxygen content and 4% plateauing for the carboxylic groups, which is in good agreement with the literature since this gas mostly accounts for physical etching^35^,^60^.

**Figure 6:**
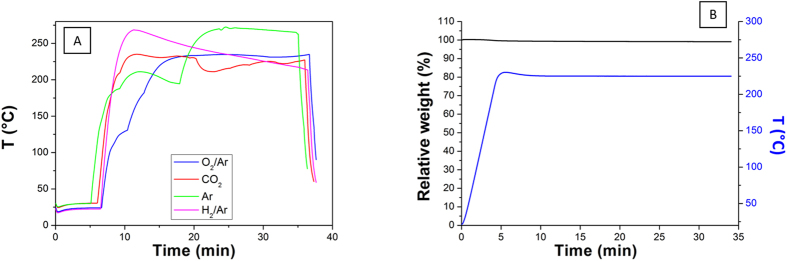
(**A**) Representative plasma temperatures for each 30-min sample showing that the temperature is almost constantly above 200 °C, and (**B**) TGA analysis in air of a reference BP.

Potential thermal damage from the plasma was also assessed. Since the plasma temperature during the treatment under the experimental conditions was found to raise above 200 °C, as shown in Fig. 6, a TGA analysis was performed on a reference BP in order to potentially rule out the decomposition of the sample as an impact factor on the Raman results. Figure 6 reveals that the original weight is almost retained up to that temperature, with less than 0.9 wt% of loss, attributed to water evaporation below 100 °C. This result suggests that the temperature is neither decomposing the structure nor affecting results from other techniques used to perform measurements for this study to evaluate type and magnitude of physical modifications occurring on the surface. Furthermore, according to the XPS patterns for the different C-O bonds introduced, since the relative content in each case across the series seems to reach a plateau this parameter can be likely considered as ineffective to vary or to tune the specific C-O bond content.

**Figure 7:**
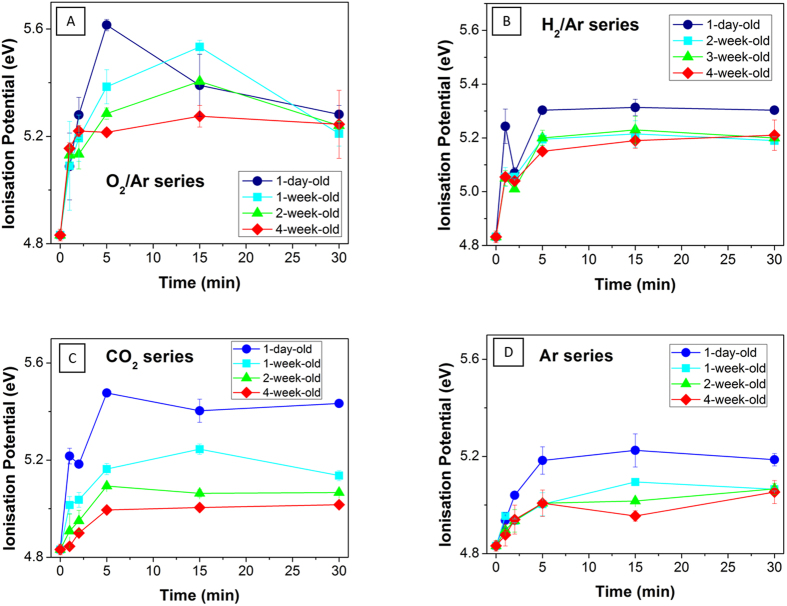
Ionisation potential as a function of treatment time (1, 2, 5, 15 and 30 min) with the following plasmas (**A**) O_2_/Ar, (**B**) H_2_/Ar (**B**), (**C**) CO_2_ and (**D**) Ar. IP values are shown for samples stored in air for 1 day, 1, 2 and 4 weeks post-plasma treatment.

The ionization potential (IP) of the CNT BPs was measured by PESA at various stages post-treatment (Fig. 7) to assess the degree of functionalisation and its temporal stability. The IP corresponds to the energy required to remove a surface atom electron from sample surface to the vacuum level^61^ and, in the case of metallic materials, is equivalent to the work function. As such the IP is highly sensitive to the state of the surface and is affected by surface contamination, the surface chemistry, surface functional groups and their density, as well as the degree of crystallinity^62^,^63^. It is therefore an excellent tool to further gauge the effects of the 4 plasma treatments under investigation here.

As seen in Fig. 7, plasma treatment led to a rapid increase in the IP over the first 5 min, regardless of the plasma type. The IP then leveled off (H_2_/Ar, CO_2_, Ar) or decreased slightly (O_2_/Ar) with further treatment duration. While all of the treatments were effective in increasing the IP, the greatest increase was observed for O_2_/Ar with an IP value of 5.6 eV after only 5 min of treatment, compared to 5.4, 5.3 and 5.2 eV for CO_2_, H_2_/Ar and Ar plasma glows, respectively. The increased work function is attributed to a combination of the introduction of oxygen functional groups at the surface and the resulting oxygen surface dipoles created, and a reduction in pi conjugation bonding (e.g. reduced sp2/sp3 ratio)^64^,^65^. CNTs functionalized with oxidative treatments, such as pure O_2_ or an acid treatment commonly exhibit increased IPs^62^. This trend is also consistent with the XPS results which showed a strong increase in hydroxyl, carbonyl and carboxylic groups at the surface for all of the plasma treatments, especially O_2_/Ar and H_2_/Ar, and a considerable decrease in the sp^2^/sp^3^ ratio and degree of surface graphitization after treatment, especially for the H_2_/Ar and CO_2_ plasma environments. The plateau observed for longer treatment durations is likely related to a saturation of the surface dipoles generated during plasma treatment and is consistent with the surface saturation of oxygen content and C-O bonds, as demonstrated by the XPS spectra.

The decay of the IP as a function of time after completing the plasma treatment was also investigated to evaluate the temporal stability of the functional groups grafted across the graphitic planes, which can potentially be affected by atomic reconfiguration (Fig. 7). Interestingly, the time dependence of the IP was found to follow a similar pattern for all the series of plasma gases tested. Although the IP decreased for all samples over time, a steady state was reached much quicker for the H_2_/Ar series (14 days) than for the O_2_/Ar series (21 days) or the CO_2_ series (28 days). In particular, the base level of the WF was found to decrease over time by 0.4 eV at 30 days post-treatment for the O_2_/Ar reactant gas, but still remained 0.38 eV above the IP of the native CNTs. This result suggests that the remaining groups are indeed permanently attached across the graphitic walls of the CNTs. The temporal stability of the treatments is also suggesting that an extended exposure to free radicals within the plasma glow may have reached an activation energy level of the graphitic plans, above which the material may interact more strongly with oxygen from air during storage after the treatment. These activated sites, generated in larger amounts with longer treatment durations and therefore being more stochastically favorable, and may indeed react with atmospheric gases to re-assemble the surface dipoles across the CNT to their original configuration. This hypothesis would also explain why a higher amount of atomic oxygen content was produced by the H_2_/Ar reacted when there while neither measurable nor significant quantities of oxygen molecules were present during the plasma treatment. These results open the way to the design of specific IP materials where the surface density and coverage of functional groups across graphitic materials such as graphene or carbon nanotubes may be simply controlled through specific plasma treatments.

This study therefore provided for the first powerful insights on the stability and time decay of functional groups generated across graphitic materials. The understanding of molecular rearrangements at the nanoscale within graphene and CNT materials is critical to develop commercially viable products and optimize chemistry as advanced platforms for a range of applications.

## Materials and Methods

### Carbon nanotube growth and BP preparation

The CNTs were grown by chemical vapour deposition (CVD) following a previously described route^66^. In short, a 1–5 nm thick iron catalyst film was deposited onto a silicon substrate bearing a thin silicon dioxide layer and a mixture of helium (95 wt%) and acetylene (5 wt%) was used as the CVD feedstock for growing CNT at a temperature between 650 and 750 °C^67^. These CNTs typically have an outer diameter of ∼10–15 nm and a length of 150–300 μm. The CNT BP membranes were processed by vacuum filtration of CNTs dispersed in 99.8% pure propan-2-ol (Aldrich 99.9%)^68^. Well-dispersed CNT suspensions were obtained by repeated sonication at intervals of 15 minutes and a power of 150 W. Vacuum filtration was performed using a 47 mm diameter Millipore filtration unit attached to line vacuum (dP = −95 kPa). The CNTs were filtered onto a poly (ether sulphone) 0.2-μm pore size Millipore membrane and then peeled off to form a self-supporting membrane. The thickness of the BPs were on the order of 20+/−2 μm. All other chemicals (polydimethylsiloxane, 2-methyl imidazole, zinc nitrate hexahydrate) and gases (Analytical air, O_2_ in Ar, H_2_ in Ar, CO_2_) used in this study were purchased from Sigma Aldrich or BOC. The composition of the gas was 28 v% O_2_ in Ar, Ar and CO_2_ of analytical grade (purity >99.99%) or 2 v% H_2_ in Ar.

### Surface plasma treatment conditions

Plasma treatments were performed on a PICO G plasma rig (Diener PICO PC SN 80001) in isotropic plasma generated by a High Frequency Generator at 13.56 MHz in a stainless steel chamber, represented in Figure S10. In this specific configuration, the active electrode is located near the ceiling of the breakdown chamber while the sample is placed in the lower, larger part below the electrode. Most of the high frequency electric power is absorbed in the clearance between the electrode and the top ceiling, which acts as the counter electrode. Products from the breakdown region spill into the lower part of the chamber to apply a uniform and isotropic treatment to the sample located there. The samples were treated at a fixed plasma chamber pressure of 0.2 mbar and a power of 80 W, while effects from the exposure were evaluated at several points in time in the following with regard to physical and chemical modifications occurring on the material surface. Plasma exposure durations were varied and the treatment time varied between 1 and 30 min. Changes in plasma temperature were recorded by means of a thermocouple. The temperature of the sample stage was held constant at 22 °C.

### Materials characterization techniques

Scanning Electron Micrographs (SEMs) were acquired on a JEOL JSM 7800F SEM at an accelerating voltage of 5 kV and a working distance of 10 mm. The samples mounted on aluminum stubs with carbon tape were not coated prior to imaging to avoid altering their physical aspect. Transmission Electron Micrographs (TEMs) were acquired on a FEG Jeol JEM2100 Lab6 TEM at 200 kV and under high vacuum. The carbon nanotube samples were gently dispersed in iso-propanol and sonicated with a bath sonicator for 5 min to suspend the CNTs. A volume of 100 μL was then dropped onto a copper grid and the samples were left to dry in air in a contained dust free chamber overnight prior to imaging.

The ionization potential was measured using Photoelectron Spectroscopy in Air (PESA – Model AC-2). In this technique UV light is used to generate photo-electrons which ionize atmospheric oxygen molecules that are accelerated towards and open counter detector. By plotting the yield (n = 0.33 or 0.5 for semiconductor or metal materials, respectively) versus energy, the ionization energy can be determined. A UV intensity of 10 nW, UV energy between 4.2–6.2 eV with 0.1 nW step, and n = 0.5, was used. For each sample at least 3 measurements were performed on different areas and the average taken.

Raman Spectroscopy was performed with a Renishaw inVia Raman Microscope, using a 514 nm laser at 10% of the nominal power. The acquisition time was 10 s and the results were based on 3 accumulations. Measurements were conducted for each sample in at least two different spots, from which an average value was calculated and the standard deviation included in the figures as error bars. The samples were tested 1 day and 1 week post treatment to investigate temporal effects. The thermogravimetric analysis was conducted on a pristine BP by a Perkin Elmer TGA 7 Thermogravimetric Analyzer. Tests were carried out at a rate of 50 °C/min in air and the temperature was kept constant at 225 °C for 30 min in order to reproduce the plasma chamber conditions.

X-ray photoemission spectroscopy (XPS) spectra were acquired at a pass energy of 160 eV with a 1 eV/step and a pressure in the analysis chamber of 5 × 10^−9^ torr with an AXIS Nova (Kratos Analytical Ltd., UK). The samples were irradiated with Al Kα radiation (*hν* = 1486.6 eV) from a mono-chromated source operating at 150 W. In a typical set up, the X-ray source is at 45 from the sample surface while the angle between the analyser and the sample surface is 90. The analysis was performed on 3 different spots on each sample, under the same experimental conditions. XPS spectra were evaluated with CasaXPS software in accordance with the experimental parameters. The C sp^2^ energy peak was calibrated at 284.5, consistent with the deconvolution of high resolution C 1s.

## Additional Information

**How to cite this article**: Merenda, A. *et al*. Assessing the temporal stability of surface functional groups introduced by plasma treatments on the outer shells of carbon nanotubes. *Sci. Rep.* **6**, 31565; doi: 10.1038/srep31565 (2016).

## Supplementary Material

Supplementary Information
