# Supplementary material for: Assessing the temporal stability of surface functional groups introduced by plasma treatments on the outer shells of carbon nanotubes
Source: Sci Rep. 2016 Aug 10;6:31565. doi: 10.1038/srep31565 (PMC4979011; doi:10.1038/srep31565)
Supplement: Supplementary Information [file srep31565-s1.doc]

Supplementary Materials for:

**Assessing the temporal stability of surface functional groups introduced by plasma treatments on the outer shells of carbon nanotubes**

Andrea Merenda^1^, Elise des Ligneris^1^, Kallista Sears^2^, Thomas Chaffraix^1^, Kevin Magniez^1^, David Cornu^3^, Jürg A. Schütz^4^, Ludovic F. Dumée^1*^

1 Deakin University, Geelong, Institute for Frontier Materials, 3216 VIC, Australia

2 CSIRO Manufacturing, Clayton - 3149 VIC, Australia

3 Institut Européen des Membranes, IEM, UMR-5635, Université de Montpellier, ENSCM, CNRS, Place Eugène Bataillon, 34095 Montpellier cedex 5 France

4 CSIRO Manufacturing, Waurn Ponds - 3216 VIC, Australia

^*^Corresponding author: Tel: +61410131312. Email: [ludovic.dumee@deakin.edu.au](mailto:ludovic.dumee@deakin.edu.au)


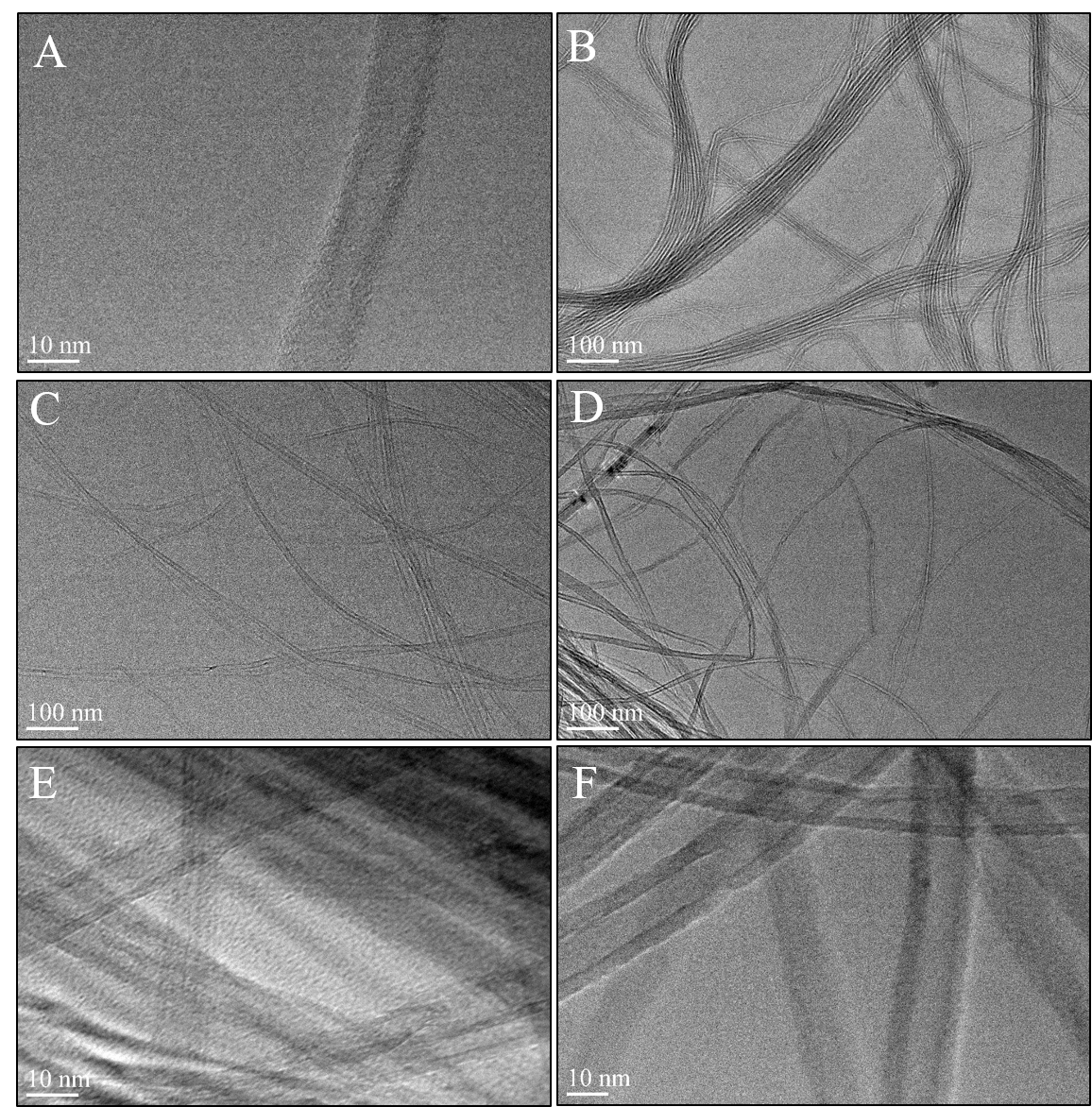


Figure S1 Transmission Electron Micrographs (TEMs) of CNTs under different conditions: plasma treated with O_2_/Ar (A) for 5 min, plasma treated with H_2_/Ar (B) for 5 min, CO_2_ (C and D) for 1 min and 30 min respectively5 min (D) and Ar (E and F) for 1 min and 30 min respectively


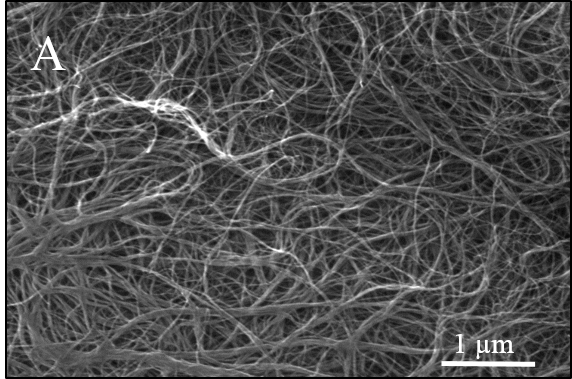


Figure S2 Scanning Electron Micrograph (SEM) of BP reference


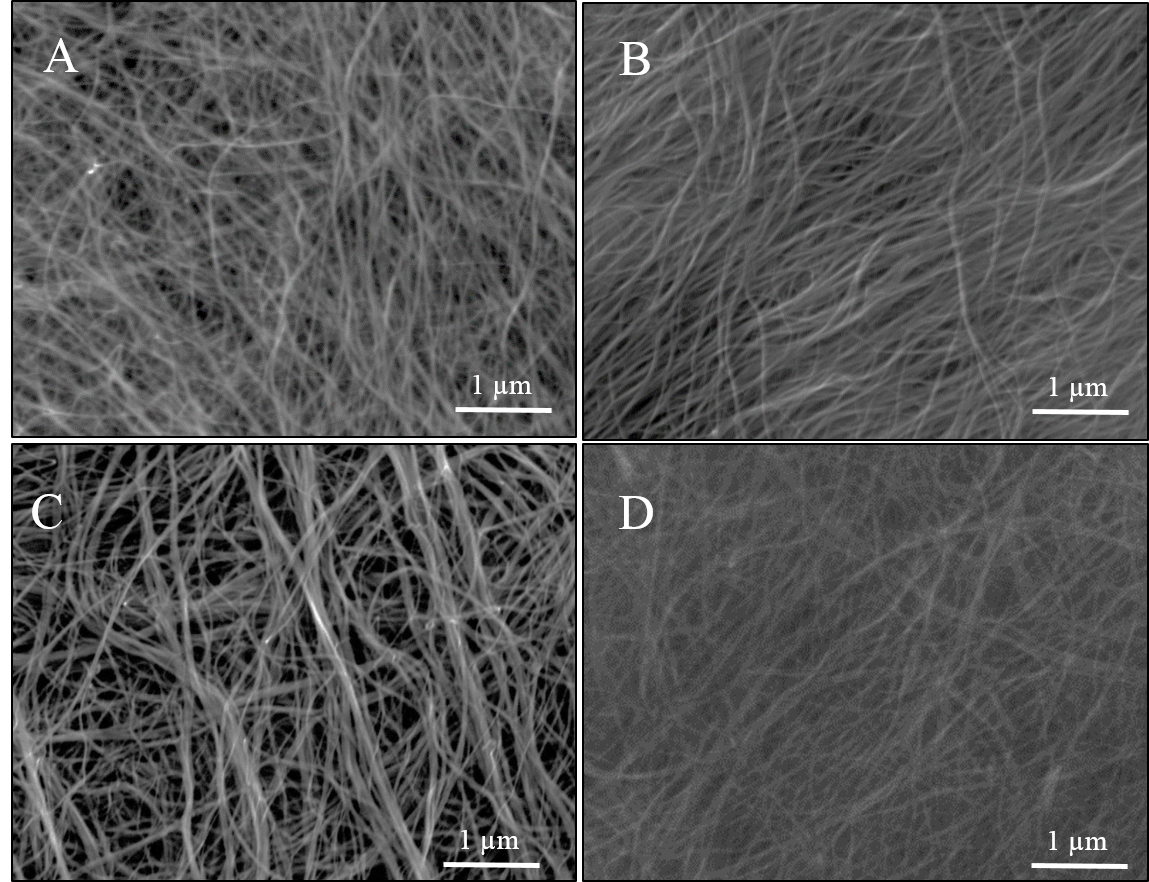


Figure S3 Scanning Electron Micrographs (SEMs) of plasma treated BPs for 1 min with O_2_/Ar (A), H_2_/Ar (B), CO_2_ (C) and Ar (D) respectively


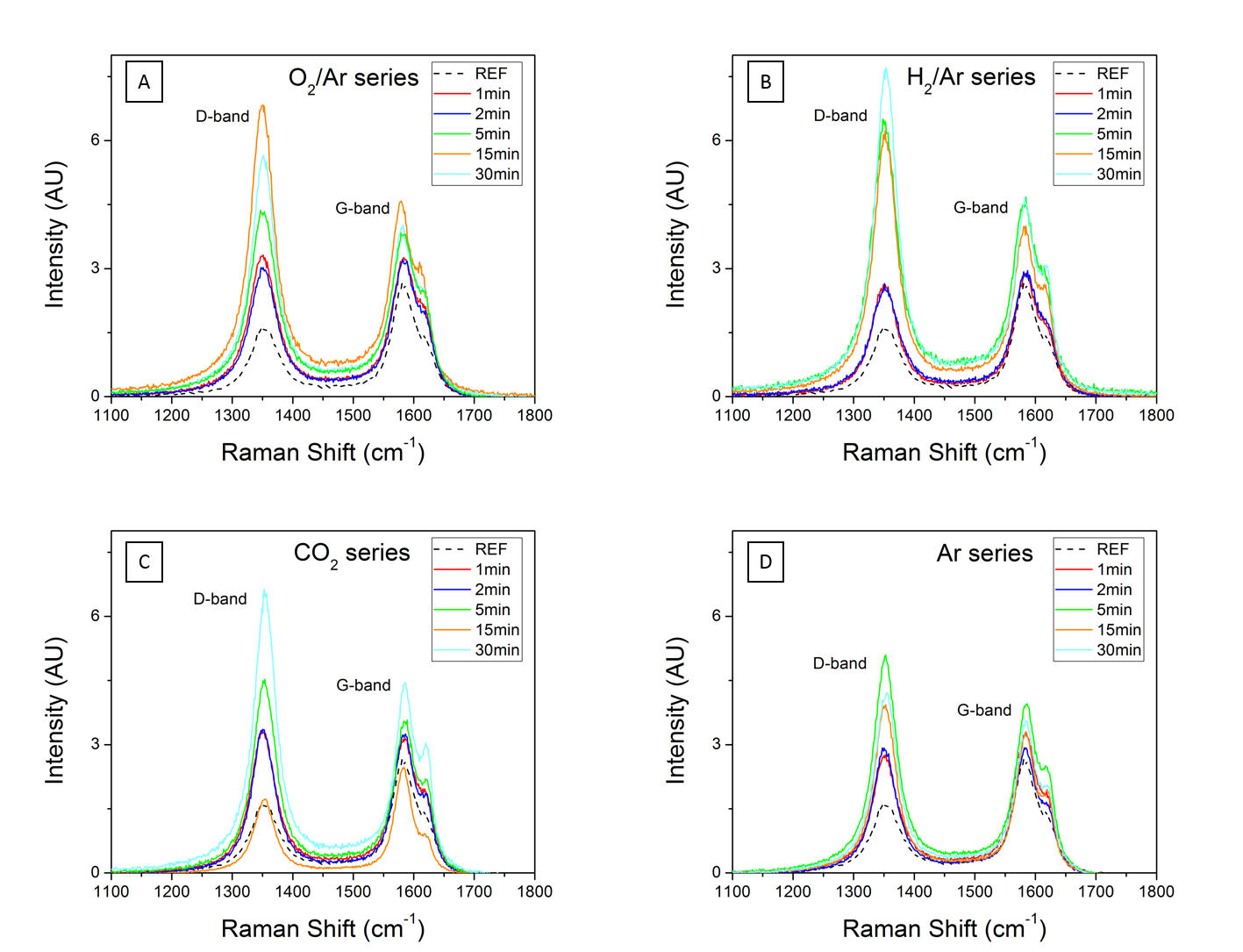


Figure S4 Raman spectra across the different series: O_2_/Ar (A), H_2_/Ar (B), CO_2_ (C), Ar (D) at different plasma treatment time, D-band and G-band in evidence. The 1-year-old series is showed, the spectra refer to the spot which was statistically closer to the Ig/Id calculated average.


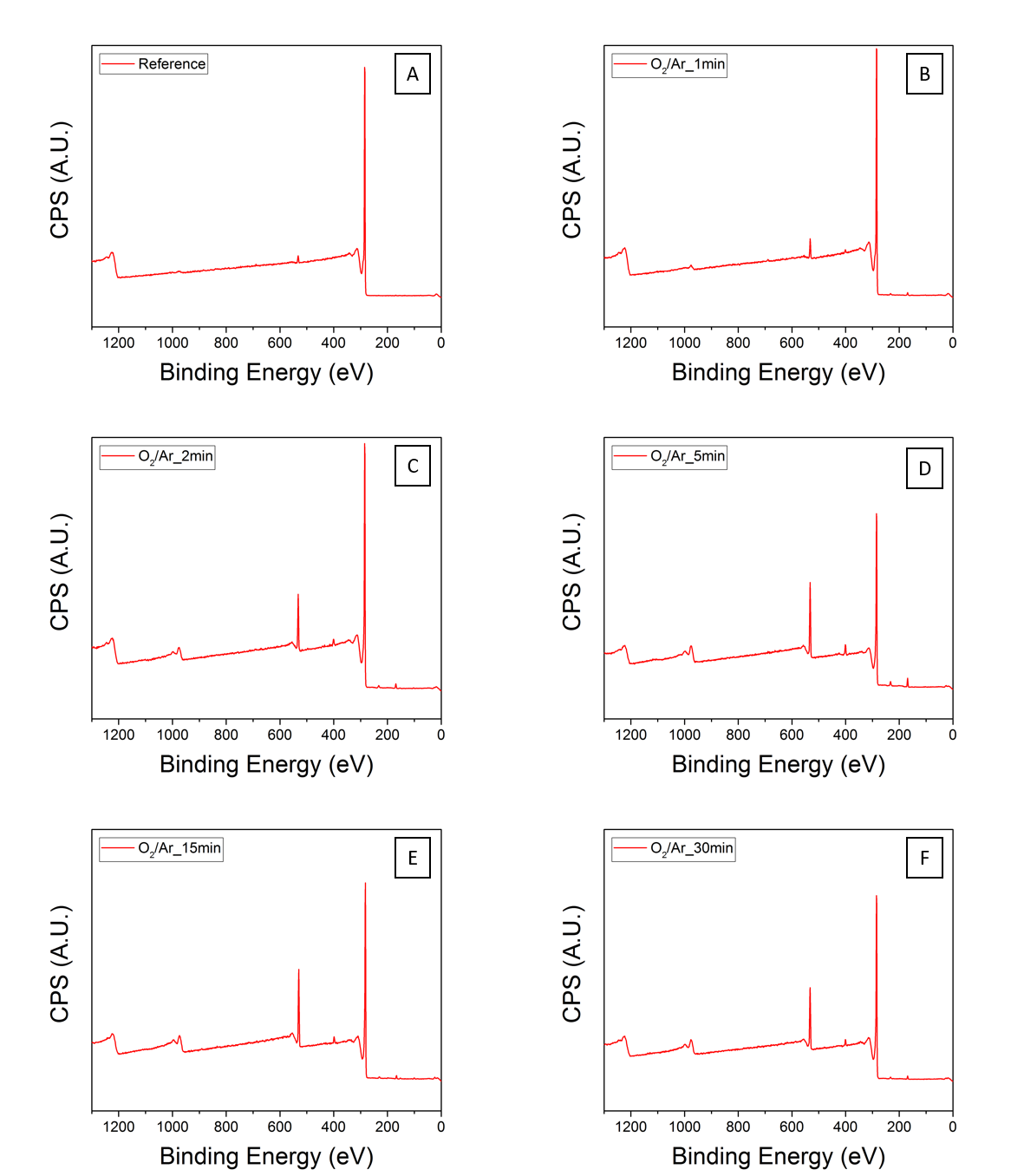


Figure S5 XPS survey spectra of O_2_/Ar series at different plasma treatment time: reference (A), 1 min (B), 2 min (C), 5 min (D), 15 min (E), 30 min (F).


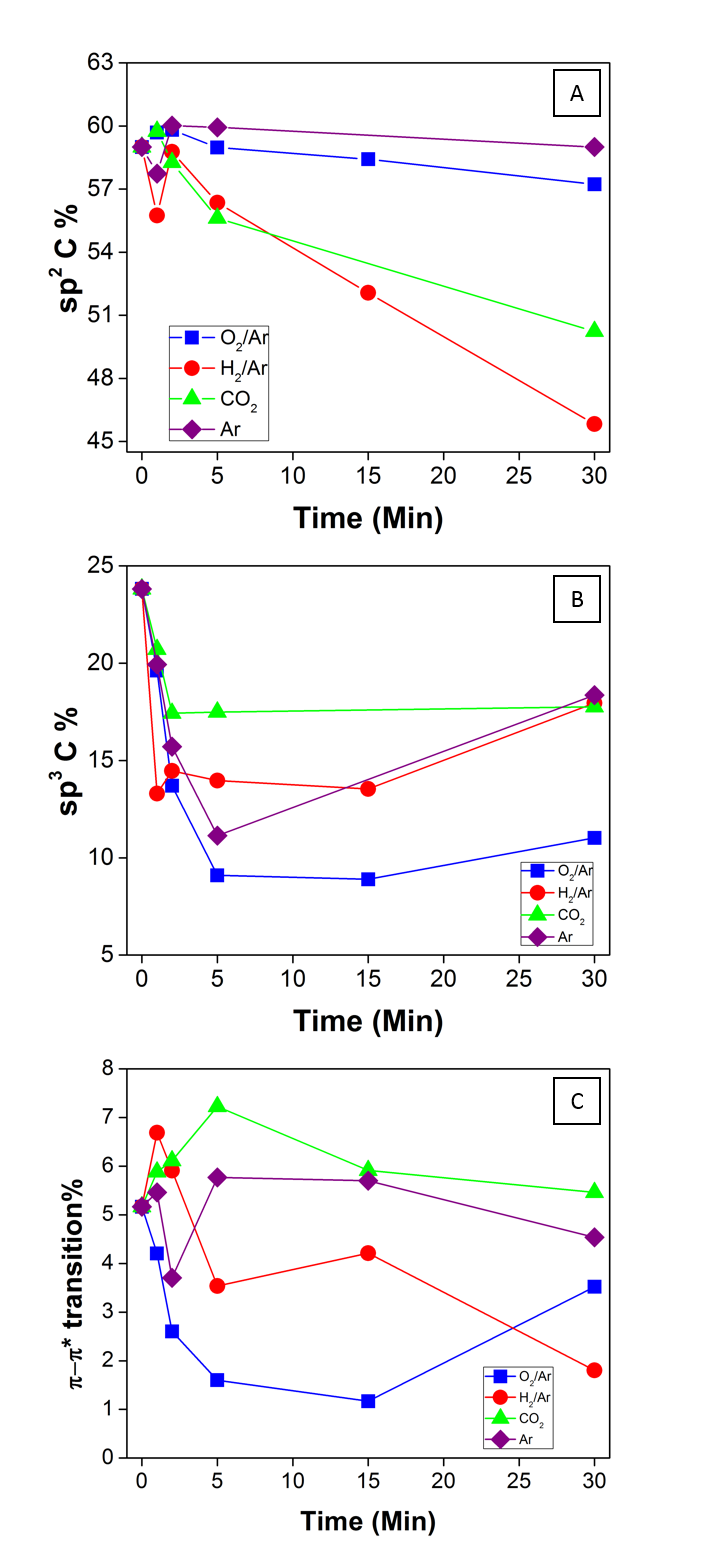


Figure S5 sp^2^ (A), sp^3^ (B) carbon content and π- π* transition (C=) evaluated by X-ray photoemission spectroscopy (XPS) across the different series: O_2_/Ar (blue), H_2_/Ar (red), CO_2_ (green), Ar (purple) at different plasma treatment time


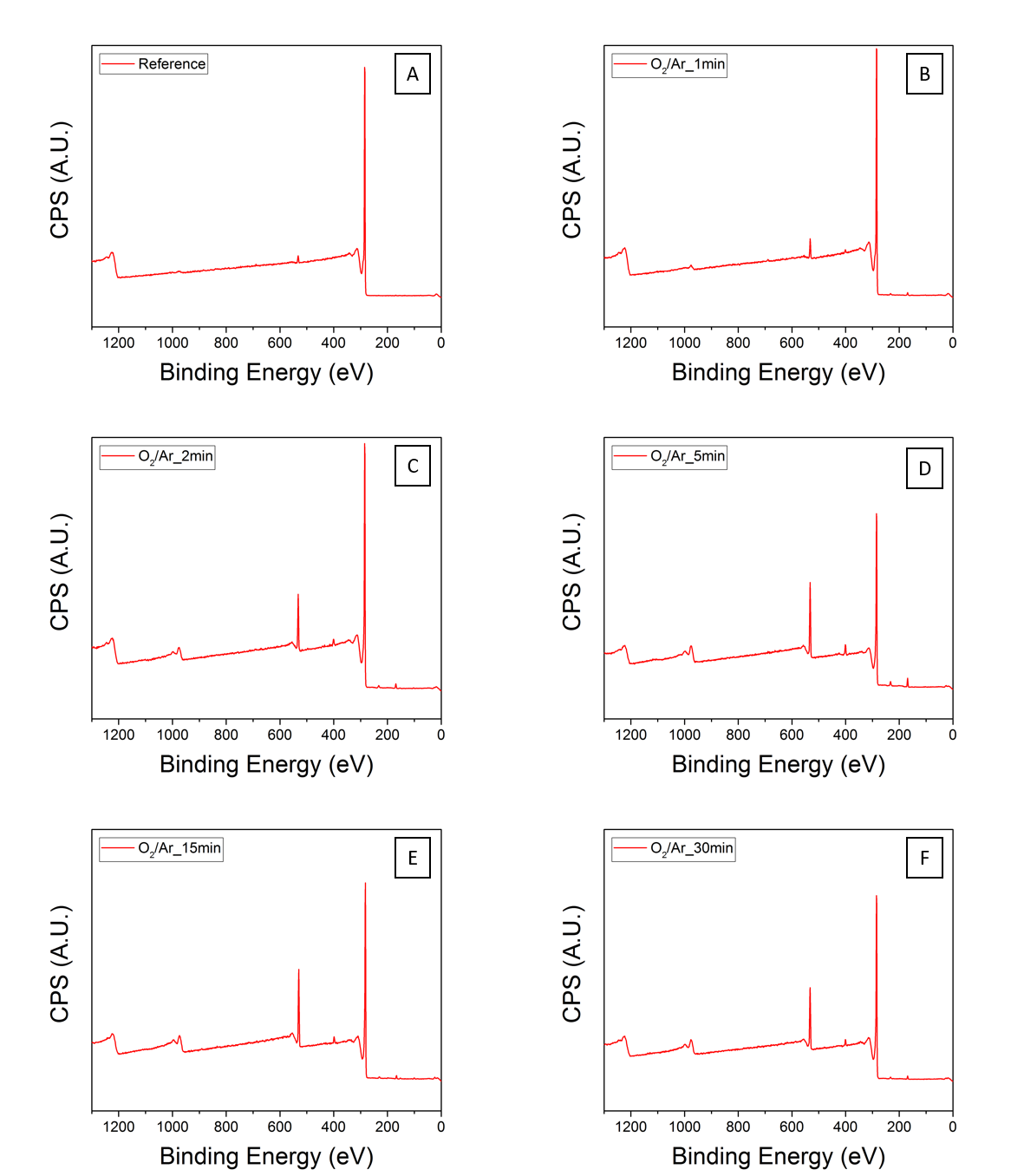


Figure S6 XPS survey spectra of O_2_/Ar series at different plasma treatment time: reference (A), 1 min (B), 2 min (C), 5 min (D), 15 min (E), 30 min (F).


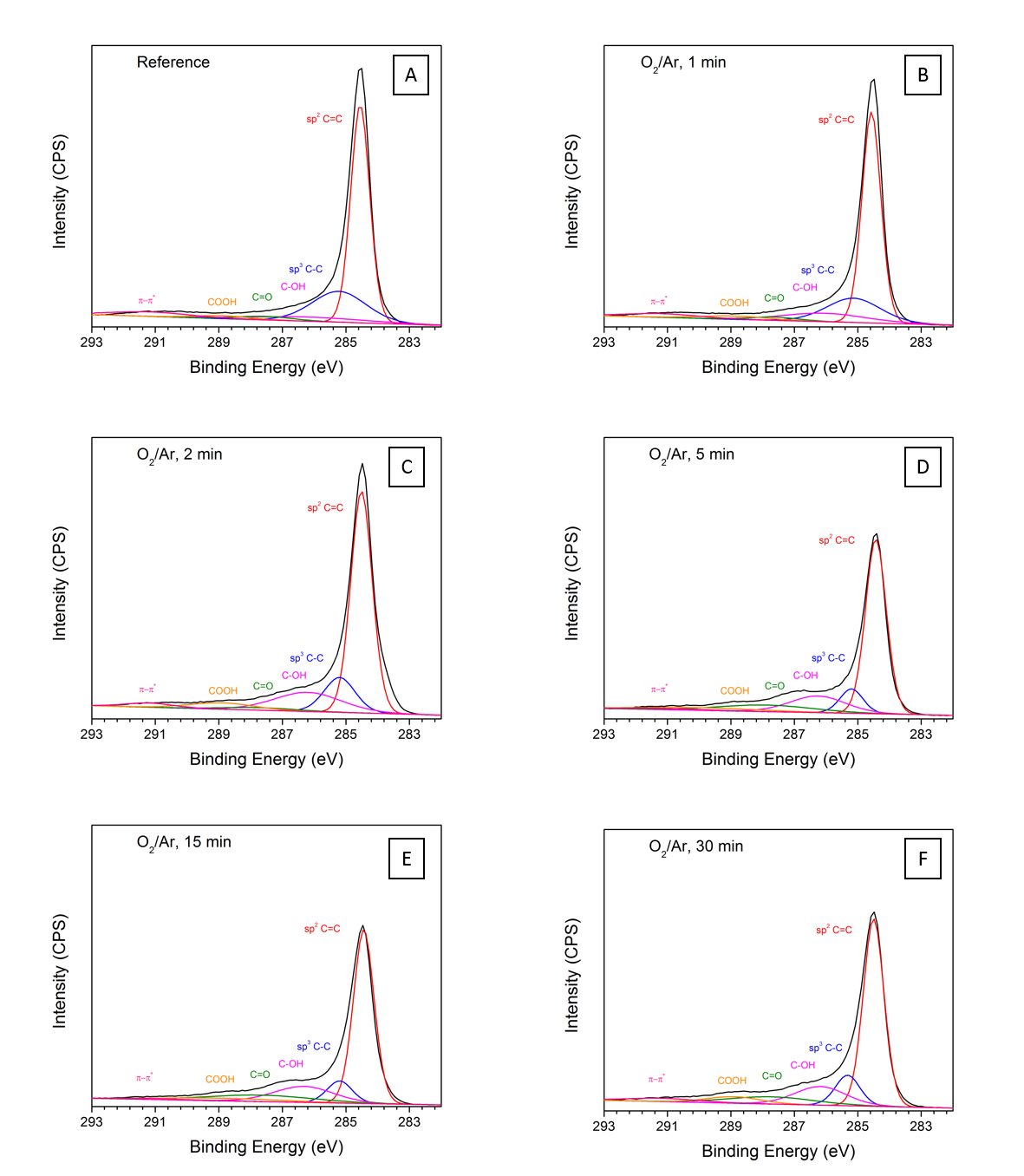


Figure S6 High resolution C1s spectra of O_2_/Ar series at different plasma treatment time: reference (A), 1 min (B), 2 min (C), 5 min (D), 15 min (E), 30 min (F).


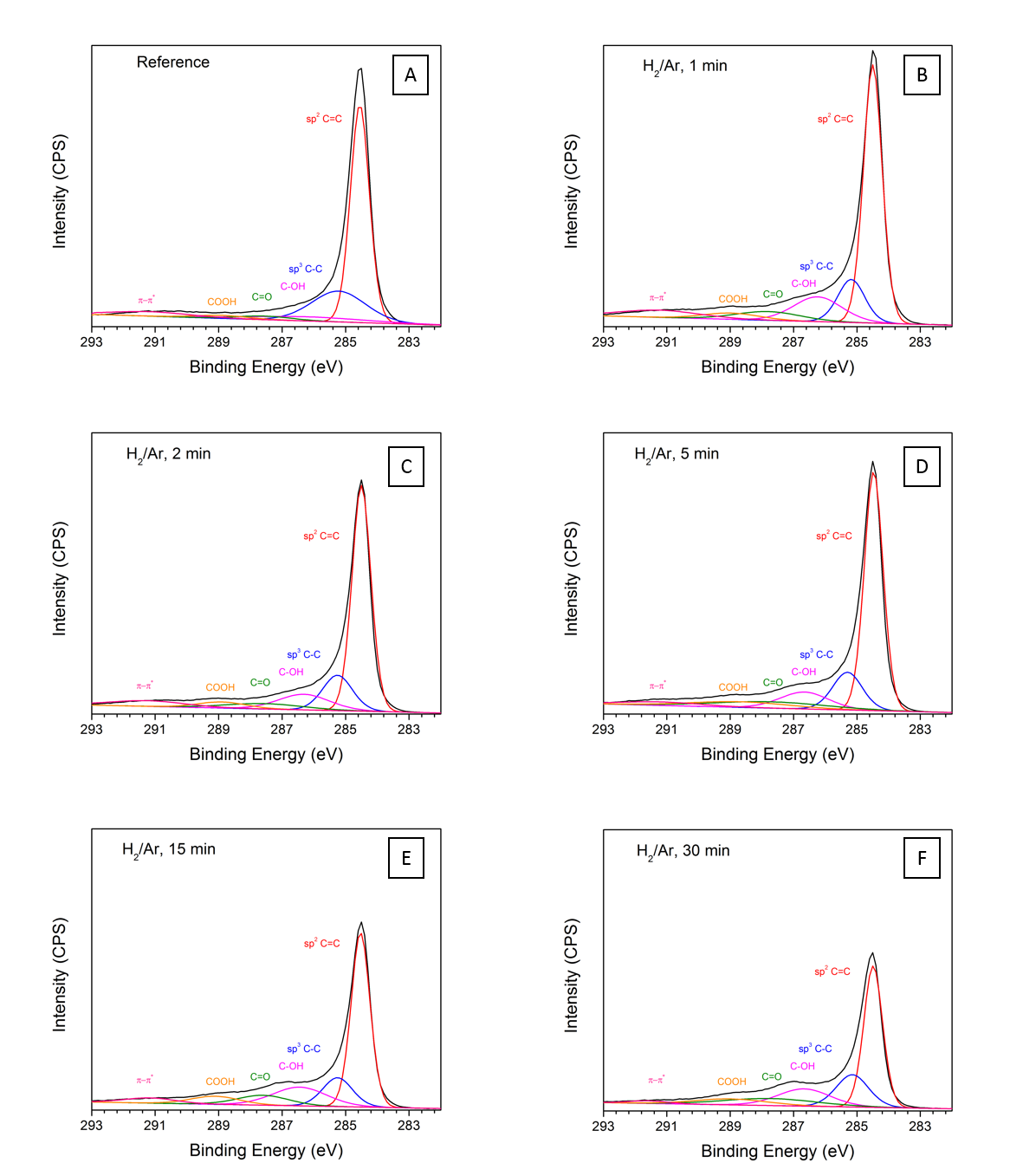


Figure S7 High resolution C1s spectra of H_2_/Ar series at different plasma treatment time: reference (A), 1 min (B), 2 min (C), 5 min (D), 15 min (E), 30 min (F).


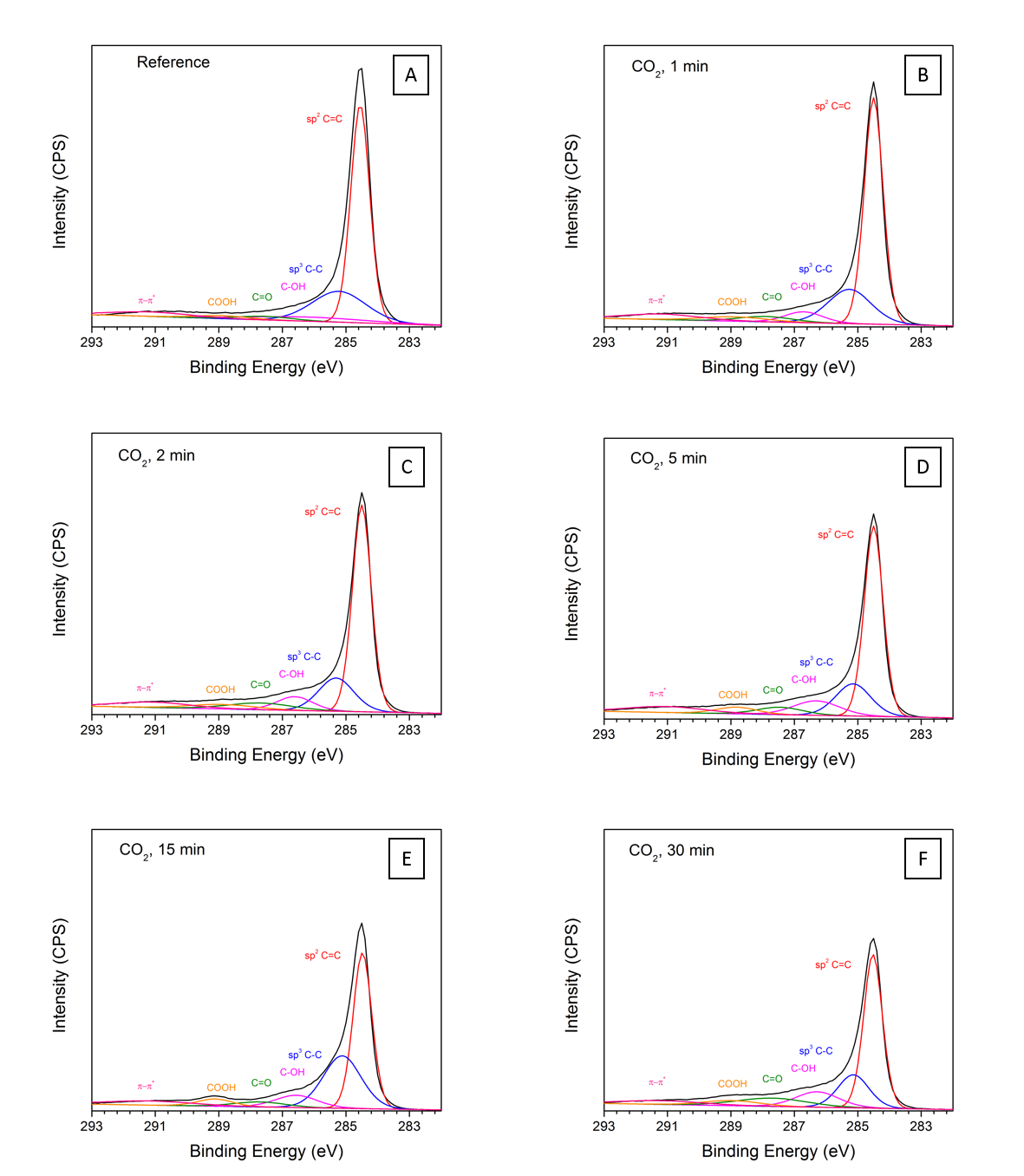


Figure S8 High resolution C1s spectra of CO_2_ series at different plasma treatment time: reference (A), 1 min (B), 2 min (C), 5 min (D), 15 min (E), 30 min (F).


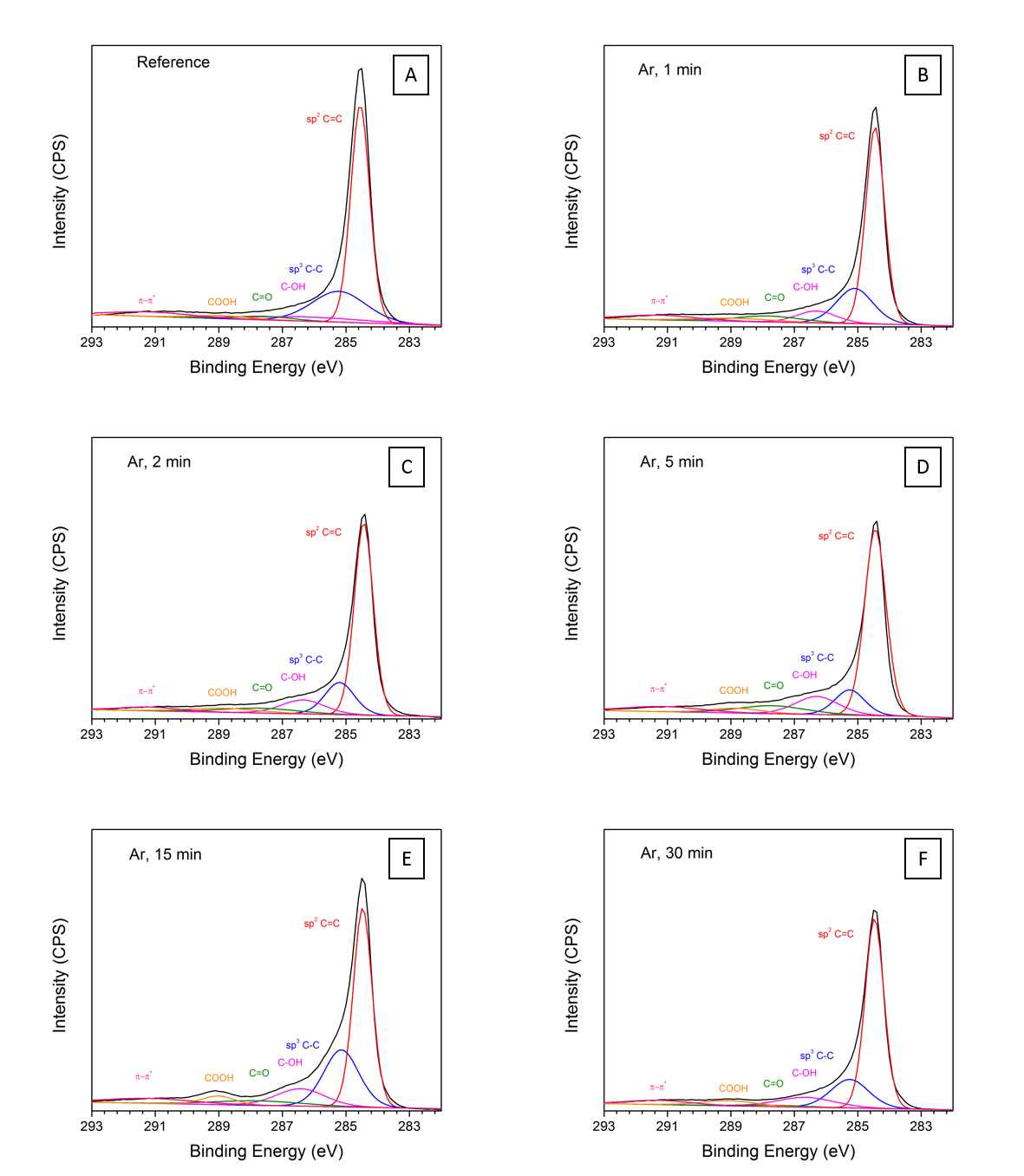


Figure S9 High resolution C1s spectra of Ar series at different plasma treatment time: reference (A), 1 min (B), 2 min (C), 5 min (D), 15 min (E), 30 min (F).


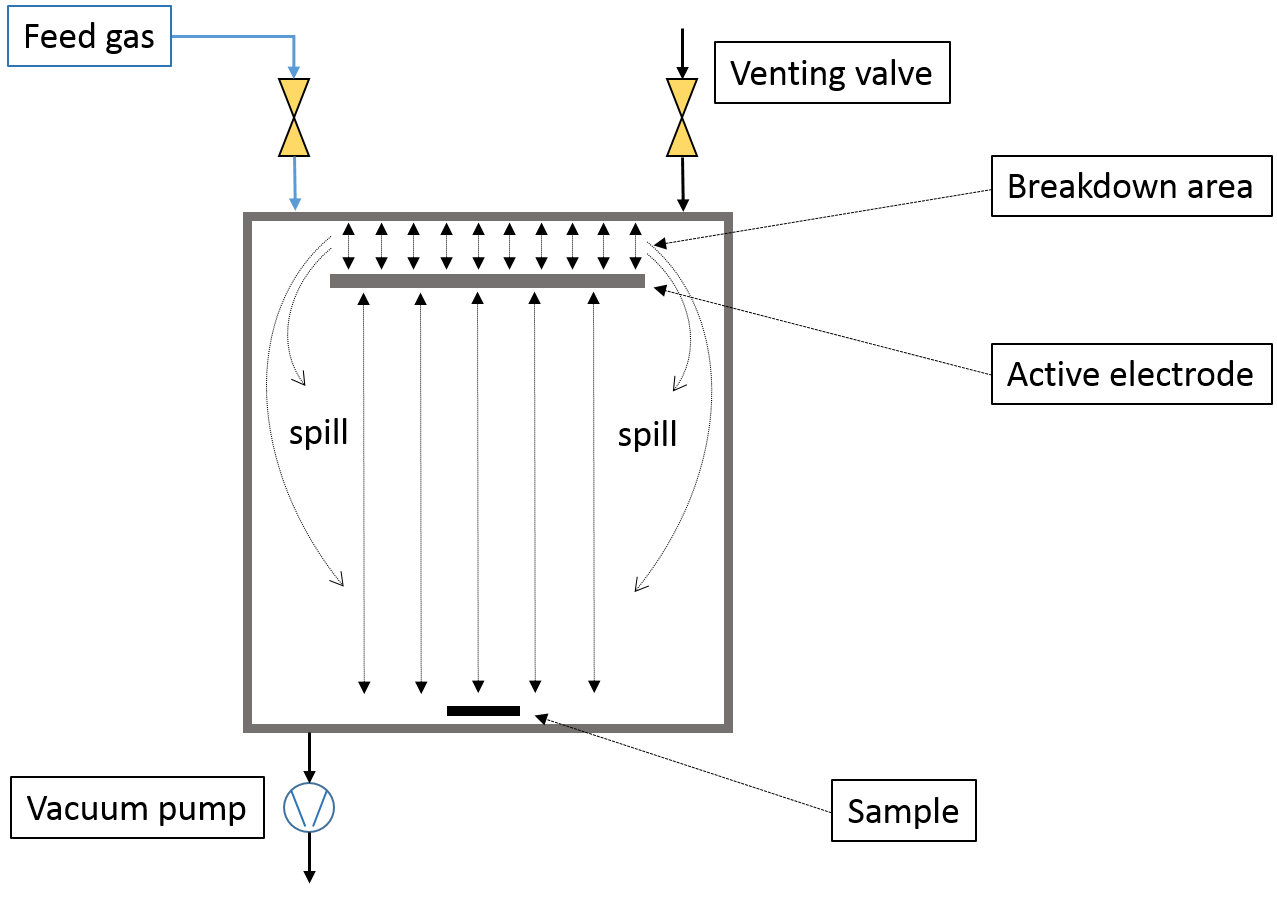


Figure S10 Schematic of plasma chamber with characteristic “spill out” and breakdown area above the active electrode.
